# Increased NMUR1 Expression in Mast Cells in the Synovial Membrane of Obese Osteoarthritis Patients

**DOI:** 10.3390/ijms231911237

**Published:** 2022-09-23

**Authors:** Ayumi Tsukada, Ken Takata, Shotaro Takano, Yoshihisa Ohashi, Manabu Mukai, Jun Aikawa, Dai Iwase, Gen Inoue, Masashi Takaso, Kentaro Uchida

**Affiliations:** 1 Department of Orthopedic Surgery, Kitasato University School of Medicine, 1-15-1 Minami-ku Kitasato, Sagamihara 252-0374, Japan; 2 Shonan University of Medical Sciences Research Institute, Nishikubo 500, Chigasaki 253-0083, Japan

**Keywords:** neuromedin, neuromedin receptors, mast cells, obese, osteoarthritis

## Abstract

Obesity is a risk factor for knee osteoarthritis (KOA). Neuromedin U (NMU) and NMU receptors (NMUR1 and NMUR2) are associated with obesity-related disorders and found in mast cells (MCs), which are elevated in osteoarthritis. However, NMU/NMUR expression was not examined in the synovial membrane (SM) or synovial MCs of obese osteoarthritis patients. We compared expression of *NMU*, *NMUR1*, *NMUR2*, and the mast cell (MC) marker, *CPA3*, in the SM of KOA patients categorized as normal weight (NW; BMI < 25 kg/m^2^, *n* = 79), overweight (OW; BMI ≥ 25 and <30 kg/m^2^, *n* = 87), and obese (OB; ≥30 kg/m^2^, *n* = 40). To study *NMU*/*NMUR* expression in MCs, we compared the MC-rich fraction (MC-RF), CD88(+) MC-RF, and CD88(−) MC-RF, extracted using magnetic isolation, with the MC-poor fraction (MC-PF). While *NMU* and *NMUR2* expression were comparable, *NMUR1* was significantly elevated in OW and OB compared to NW. Moreover, *CPA3* levels were significantly greater in OB than NW. *NMUR1* and *CPA3* expression were significantly higher in both the CD88(+) and CD88(−) MC-RF than MC-PF. Therefore, *NMUR1* expression was elevated in the SM of OB KOA patients, and its expression was found in MCs. Further investigation to analyze the NMU/NMUR1 pathway in MC may provide a link between obesity and KOA pathology.

## 1. Introduction

Research suggests that being obese is associated with an elevated prevalence and occurrence of osteoarthritis (OA) in both weight-bearing and non-weight-bearing joints [1,2]. Such evidence implies that factors other than mechanical loading are involved in the link between obesity and OA. These factors could additionally play a role in OA pathology. To date, however, these factors and their related mechanisms remain to be elucidated.

According to several studies, synovial neuropeptides may contribute to OA pathology [3,4,5,6,7]. Neuromedin U (NMU) is one such example. This bioactive peptide was initially extracted from the spinal cord of pigs [8] and forms part of the NMU system, which also comprises the NMU receptors, NMUR1 and NMUR2. Together, these components of the NMU system play a role in multiple physiological functions such as inflammation, stress responses, circadian rhythmicity, and feeding behavior [9,10,11,12,13]. Experiments using genetic ablation or overexpression of NMU have demonstrated the presence of crosstalk between the NMU system and obesity-related pathological factors [14,15]. NMU was also shown to promote autoantibody-mediated arthritis in mice [16]. To the contrary, NMU was reported to be a suppressor of this pathology in a murine collagen-induced arthritis (CIA) model [17]. However, expression levels of NMU/NMURs were not examined in the synovium of obese OA patients.

*NMU* mRNA was observed in antigen-presenting cells, such as monocytes and dendritic cells. Meanwhile, *NMUR1* mRNA is present in T cells, natural killer cells, eosinophils, and mast cells (MCs) [10,12,13,18]. In addition to being found in the synovial membrane (SM), MCs are elevated in patients with rheumatoid arthritis (RA) [19,20,21] and OA [22,23,24], suggesting that MCs could constitute a crucial component of the mechanism underlying both acute and chronic inflammation. More recent studies have demonstrated a potential association between MCs and knee OA (KOA) severity [25,26]. Given that we also previously reported increased MC marker expression in obese KOA patients [6,27,28], we hypothesized that MCs may contribute to the NMU/NMURs system in the osteoarthritic synovium of obese individuals.

Complement receptors (CRs) play an important role in innate immune defense and local inflammation [29]. Complement component 5a (C5a) and C5a receptor (CD88) signaling play an important role in MC activation via granulation [30]. A previous study reported that C5a-receptor (CD88)-positive MCs exist in the SM of OA and rheumatoid arthritis and that the number of these cells was increased in RA [31]. In addition, increased C5a was found in the serum of obese children [32]. Therefore, investigation of NMU/NMURs expression in MC, particularly CD88(+) MC, may be important in obese KOA pathology.

Here, we studied the expression of *NMU*, *NMUR1*, *NMUR2*, and the MC marker, *CPA3*, in the synovium of obese OA patients and examined whether NMU/NMUR expression is found in synovial CD88(+) and CD88(−) MCs subsets.

## 2. Results

### 2.1. Patient Features According to BMI

The patients’ clinical features are presented in Table 1. Those categorized as overweight (OW) and obese (OB) were significantly younger than those in the normal-weight (NW) group (*p* = 0.023 and *p* = 0.010, respectively). In contrast, the percentage of patients with Kellgren and Lawrence grade 2, 3, and 4 (*p* = 0.675) KOA was not different among the BMI groups.

### 2.2. Synovial Membrane Levels of NMU/NMURs and CPA3 by BMI

**Figure 1:**
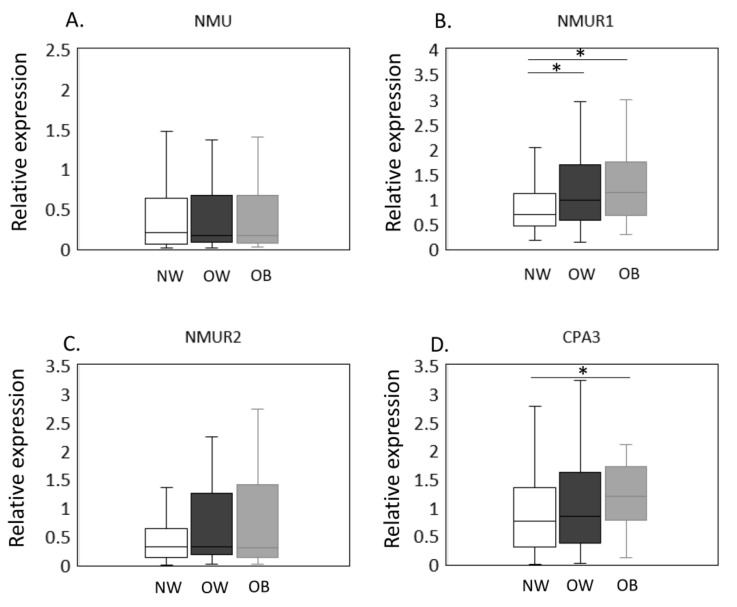
Expression of *CPA3* and *NMU*/*NMURs* in the synovial membrane of normal-weight, overweight, and obese groups. The expression of *NMU*/*NMURs* and *CPA3* mRNA in NW, OW, and OB groups was estimated by qPCR (**A**–**D**). *NMU* (**A**), *NMUR1* (**B**), *NMUR2* (**C**), and *CPA3* (**D**) mRNA expression in the synovial membrane of normal-weight (NW, *n* = 79), overweight (OW, *n* = 87), and obese (OB, *n* = 40) patients with knee osteoarthritis. Gene expression is presented in box and whisker plots, showing the median, 25th, and 75th percentiles and range. * *p* < 0.05.

The expression of *NMU*/*NMURs* and *CPA3* mRNA in NW, OW, and OB groups was estimated by qPCR (Figure 1A–D). No significant difference was observed in *NMU* (*p* = 0.948, Figure 1A) expression among the BMI groups. In contrast, NMUR1 levels were significantly greater in OW and OB than NW individuals (OW, *p* = 0.023; OB, *p* = 0.010, Figure 1B). *NMUR2*, however, was comparable across BMI groups (*p* = 0.327, Figure 1C). Meanwhile, *CPA3* expression was significantly elevated in OB compared to NW patients (*p* = 0.020; Figure 1D) but was not different between NW and OW patients (*p* = 0.872, Figure 1D).

### 2.3. Comparison of NMU/NMUR and CPA3 Expression between NW and OB Groups in a Propensity Score-Matched Cohort

**Figure 2:**
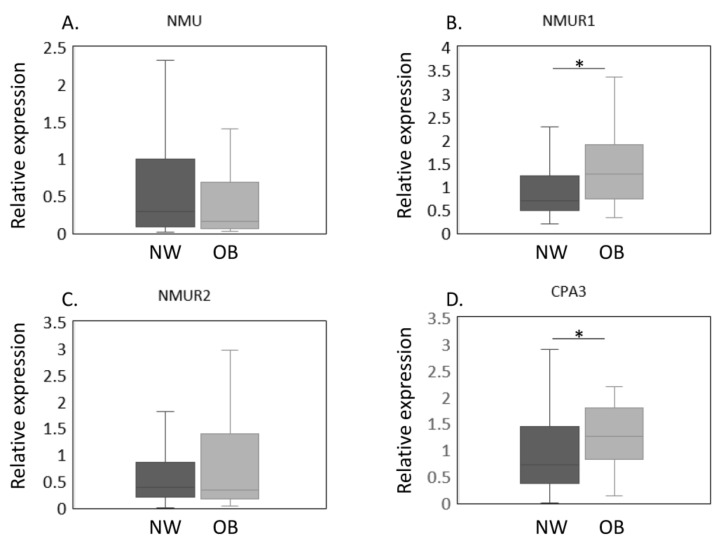
Expression of *NMU*/*NMURs* and *CPA3* in normal-weight and obese groups after propensity score matching. The expression of *NMU*/*NMURs* and *CPA3* mRNA in normal-weight (NW, *n* = 38) and obese (OB, *n* = 38) groups in a propensity score-matched cohort was estimated by qPCR (**A**–**D**). The expression of *NMU* (**A**), *NMUR1* (**B**), *NMUR2* (**C**), *CPA3* (**D**) mRNA in the synovial membrane of NW and OB patients with knee osteoarthritis after propensity score matching. Gene expressions are presented in box and whisker plots, showing the median, 25th, and 75th percentiles and range. * *p* < 0.05.

The expression of *NMU*/*NMURs* and *CPA3* mRNA in NW and OB groups in a propensity score-matched cohort was estimated by qPCR (Figure 2A–D). Our results thus far showed that OB patients had higher *NMUR1* and *CPA3* expression and were significantly younger than NW patients. To eliminate the effect of age on gene expression, we conducted a propensity score analysis to create a matched cohort. The patients’ clinical characteristics after the propensity score analysis are shown in Table 2. Both age and the proportion with patients with Kellgren and Lawrence grade 2–4 were similar between NW and OB patients (age, *p* = 0.429; proportion with Kellgren and Lawrence grade 2–4, *p* = 0.432). Similarly, no significant differences were noted in *NMU* levels (*p* = 0.448, Figure 2A). In contrast, *NMUR1* expression was significantly elevated in OB compared with NW patients (*p* = 0.012, Figure 2B). While *NMUR2* expression did not differ among the BMI groups (*p* = 0.905, Figure 2C), *CPA3* levels were significantly greater in the OB than in the NW group (*p* = 0.023, Figure 2D).

### 2.4. Expression of Synovial NMU/NMURs and CPA3 in Non-MC and MC Fractions

**Figure 3:**
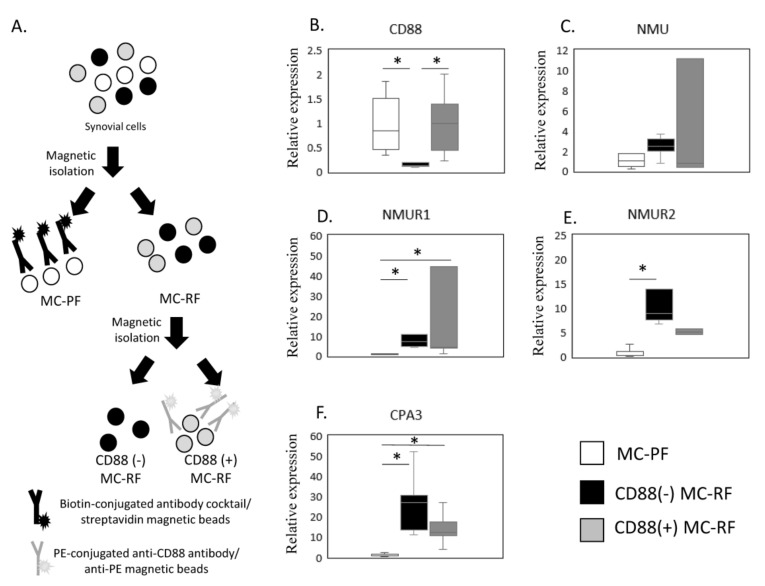
Expression of *NMU*/*NMURs* and *CPA3*. (**A**) Schematic showing the process used to isolate the mast cell (MC)-poor fraction (MC-PF; THY-1+, CD3+, CD14+, or CD19+CD235+) and CD88(−) and CD88(+) MC-rich fractions (MC-RFs; THY-1-CD3-CD14-CD19-CD235-). MC-RF and MC-PF were magnetically separated from other synovium-derived cells using biotin-conjugated antibody cocktails and streptavidin beads. Subsequently, the MC-RF was further divided into CD88(−) and CD88(+) MC-RFs using PE-conjugated anti-CD88 antibody and anti-PE beads. The expression of NMU/NMURs and CPA3 mRNA in non-MC and CD88(−) and CD88(+) MC fractions was estimated by qPCR (**B**–**F**). *CD88* (**B**), *NMU* (**C**), *NMUR1* (**D**), *NMUR2*(**E**), and *CPA3* (**F**) expression levels in MC-PF and CD88(−) and CD88(+) MC-RF derived from the synovial membrane of obese KOA patients (*n* = 5). * *p* < 0.05.

The expression of *NMU*/*NMURs* and *CPA3* mRNA in non-MC and CD88(−) and CD88(+) MC fractions was estimated by qPCR (Figure 3A–F). Given that *NMUR1* and *CPA3* were elevated in the SM of OB patients, we next examined *NMU*/*NMURs* expression in MCs by comparing the CD88(−) MC-rich fraction (MC-RF) (Figure 3A) and CD88(+) MC-RF with the MC-poor fraction (MC-PF), which we isolated from 5 SM samples from obese KOA patients (Figure 3B–F). We confirmed that CD88(+) MC-RF showed significantly higher expression of *CD88* than the CD88(−) MC-RF (*p* = 0.011, Figure 3B). While there were no differences in NMU expression among the fractions (*p* = 0.247, Figure 3C), both CD88(−) MC-RF and CD88(+) MC-RF expressed significantly higher levels of *NMUR1* (*p* = 0.011 and *p* = 0.027, respectively, Figure 3D) than the MC-PF. Additionally, *NMUR2* levels were significantly greater in the CD88(−) MC-RF than the MC-PF (*p* = 0.004, Figure 3E). Both CD88(−) and CD88(+) MC-RFs expressed significantly higher levels of *CPA3* (*p* = 0.011 and *p* = 0.027, respectively, Figure 3F) than the MC-PF.

## 3. Discussion

This study in patients with KOA found that *NMUR1* expression was elevated in the SM of OB compared to NW individuals. Further, *NMUR1* expression was detected in MC fractions, suggesting that MCs may contribute to the NMU/NMUR system in the osteoarthritic synovium of obese patients.

According to a recent report, low-grade inflammation, or metainflammation, plays a role in the pathogenesis of obesity and obesity-related diseases [33]. OB and OW individuals with KOA exhibit greater synovial inflammation than NW individuals with KOA [34]. Evidence suggests that NMU plays a role in inflammatory conditions. For example, NMU-deficient mice show lower levels of interleukin (IL)-6 secretion from macrophages [13,35]. Additionally, interaction between NMU and NMUR1 was shown to induce the release of IL-4, -5, -6, -10, and -13 in mouse helper T cell lines [36]. We found that mRNA levels of *NUMR1* were increased in the SM of OW and OB patients, suggesting that increased *NUMR1* expression in the SM may contribute to obesity-related synovial pathology. To the contrary, NMU was shown to suppress autoantibody-mediated arthritis in a murine collagen-induced arthritis model. NMU-23 administration induced the expansion of innate lymphoid cells and elevated eosinophil, IL-4, IL-5, and IL-13 expression in the joint of CIA mice [17]. As MCs contribute to arthritis by cytokine secretion, a further investigation of the proteins secreted by NMU-stimulated MCs may reveal the association between obese and KOA pathology.

A previous study reported that, compared to NW individuals, OW and OB patients undergo TKA at a younger age [6]. Consistent with this, we also noted that those in the OB group were significantly younger than those in the NW group. Furthermore, almost all synovial samples were obtained from patients with late-stage OA undergoing TKA. Given that an individual’s gene expression profile can change with age, we performed a propensity score analysis to eliminate the effect of age on gene expression and found that similar to before propensity score matching, *NUMR1* and *CPA3* expression were significantly higher than in OB than NW patients. Therefore, our findings suggest that elevated *NMUR1* and *CPA3* expression is associated with obesity but not age.

Many immune cell types engaged in obese OA pathology are present in the SM [37]. NMU and NMUR1 expression were observed in several immune cell types [9,10,13,18,36], including antigen-presenting cells such as monocytes and dendritic cells for NMU [13,36], and T cells, natural killer cells, eosinophils, and MCs for NMUR1 [9,10,13,18]. As increased levels of the MC marker, *CPA3*, were observed in the SM of obese KOA patients, we investigated the expression of *NMU*/*NMURs* in MCs. Our results suggest that *NMUR1* is expressed in synovial MCs and that elevated *NMUR1* may reflect an increase in MCs in the SM. Furthermore, given that previous studies have identified CD88(+) and CD88(−) MC subsets in SM, skin, and lung [31,38,39], and elevated levels of CD88(+) MC in the SM of individuals with RA compared to OA [31], we additionally compared NMU/NMUR in CD88(+) and CD88(−) MC-RFs. Both CD88(+) and CD88(−) MC-RFs isolated from the SM of KOA patients showed high expression of *CPA3* and *NMUR1*. According to a prior study in mice, activation of the NMU/NMUR1 pathway in MCs results in degranulation and neutrophil infiltration [40]. Additionally, MCs and their degranulation products were observed in the SM and synovial fluid of KOA patients [26]. Moreover, the number of synovial MCs correlates with the KOA patients’ synovitis score [25]. As CD88 expression in MCs contributes to granulation [30], this evidence suggests that NMU/NMUR1-mediated activation of MCs, particularly CD88(+) MC subsets, may contribute to the synovial pathology in obese KOA patients.

While NMUR1 expression was predominantly reported in peripheral tissues, NMUR2 was mostly observed in the central nervous system (CNS) [10,41,42,43,44,45,46,47,48], specifically in the hypothalamus, hippocampus, and spinal cord [43,44]. A previous study reporting NMUR2 expression in astrocytes and microglia in the mouse hippocampus suggested that NMU could regulate inflammation in the CNS. However, there are also some reports of NMUR2 expression in peripheral tissues, including in the gastrointestinal and genitourinary tracts, with high concentrations observed in the testis [47,49]. In the present study, we noted *NMUR2* mRNA in the SM, with particularly high levels in CD88(−) MCs. However, as *NMUR2* did not differ among the BMI groups, we propose that NMUR2 may play a limited role in obesity-related synovial pathology.

There were several limitations in this study. First, we showed that MC fractions highly expressed *NMUR1* compared to the non-MC fraction. However, MC-PF isolated by magnetic beads contained a mixed cell population, including fibroblasts, macrophages, B cells, and T cells. The comparison of NMUR1 expression in MC and particular cell types requires isolation using a cell sorter. Second, the role of NMU/NMUR1 on synovial MCs remains unclear. Finally, the reason *NMUR2* expression was detected mainly in the CD88(−) MCs remains unclear.

## 4. Materials and Methods

### 4.1. Patients and Methods

All participants received total knee arthroplasty (TKA) at our hospital, during which time SM samples were extracted. In total, we extracted 206 SM samples from female patients with radiographic KOA. We promptly froze a small portion of each sample in liquid nitrogen and stored it at −80 °C in preparation for RNA extraction. Samples extracted from five obese KOA patients were used to examine *NMU*/*NMUR* expression in MCs.

The study protocol was approved by our institutional Ethics Review Board (approval number: KMEO B19–259). Written informed consent was obtained from all subjects for participation and the extraction of their synovial tissue one day before TKA. This study complied with the principles of the Declaration of Helsinki.

We grouped the patients according to the World Health Organization’s body mass index (BMI) definitions as follows: normal-weight (BMI < 25 kg/m^2^, *n* = 79), overweight (BMI ≥ 25 and <30 kg/m^2^, *n* = 87), and obese (≥30 kg/m^2^, *n* = 40). Expression levels of *NMU*/*NMURs* and the MC marker, *CPA3*, in the SM determined using real-time PCR were compared between pairs of the BMI groups.

### 4.2. qPCR

SM samples in TRIzol reagent (Invitrogen, Carlsbad, CA, USA) were homogenized using the polytron homogenizer. The samples were subsequently lysed in 1 mL of TRIzol mixed with 0.2 mL chloroform and vortexed for 30 s before being transferred to a MaXtract high-density tube (Qiagen, Valencia, CA, USA). Following a centrifugation step (12,000 rpm, 5 min), the resulting aqueous phase was mixed with an equal volume of isopropanol containing a precipitation carrier (Ethachinmate; Nippon Gene, Tokyo, Japan). After removing the supernatant, the RNA pellet was rinsed with 75% ethanol and subjected to centrifugation (15,000 rpm, 4 °C, 5 min). After removing the supernatant, the RNA pellet was left to dry before dissolving in RNase-free water. The total RNA concentration was determined with a spectrophotometer (Denovix, Tokyo, Japan). An OD 260/280 ratio greater than 1.8 was used for qPCR analysis. We also confirmed that gel electrophoresis showed clear bands of 28S and 18S. A 1-μg amount of the total RNA was subjected to a cDNA synthesis procedure using Superscript III based on the manufacturer’s protocol (Invitrogen). The qPCR procedure we adopted using SYBR Green is published in detail elsewhere [21,50]. The qPCR primer sequences are provided in Table 3. Gene expression (Gene/*GAPDH*) was determined using the delta-delta CT method. Relative expression was calculated when the average gene expression (Gene/*GAPDH*) level in the NW group was 1.

### 4.3. Expression of NMU and NMURs in MCs

To evaluate *NMU*/*NMUR* expression in MCs, we extracted an MC-rich fraction (MC-RF) and MC-poor fraction (MC-PF) from 5 SM samples from obese KOA patients using magnetic isolation [27,28]. As distinct CD88(+) and CD88(−) MC subsets were previously found in SM samples taken from patients with OA and RA [31], we further divided the MC-RF into CD88(+) and CD88(−) MC-RFs.

To obtain these MC-RFs, fresh SM samples were promptly placed in a collagenase solution overnight for collagenase digestion. The following day, a portion of the cells extracted by this process were used to confirm cell viability by PI staining (cell viability, >90%). The remaining cells were incubated for 30 min at 4 °C with biotin-conjugated anti-THY-1 (synovial fibroblast marker), anti-CD3 (T lymphocyte marker), anti-CD14 (monocyte/macrophage marker), anti-CD19 (B lymphocyte marker), and anti-CD235a (erythroid cell marker) antibodies according to our previous studies [6,27,28]. All biotin-conjugated antibodies were purchased from BioLegend (San Diego, CA, USA). After rinsing twice with PBS, the cells were exposed to streptavidin-conjugated magnetic particles (BD Biosciences, San Jose, CA, USA) and placed into a magnetic separation system (BD Biosciences) for separation into negative (MC-RF; THY-1-, CD3-, CD14-, CD19-, and CD235-) and positive (MC-PF; THY-1+, CD3+, CD14+, CD19+, CD235+) fractions. Subsequently, the MC-RF was reacted with PE-conjugated anti-CD88 antibody (BioLegend) for 30 min at 4 °C. After rinsing twice with PBC, the cells were exposed to anti-PE magnetic particles (BD Biosciences, CA, USA) for separation into a CD88-negative fraction (CD88(−) MC-RF) and positive fraction (CD88(+) MC-RF). The cells in the MC-RF and CD88(−) MC-RF were then subjected to qPCR to examine *NMU*/*NMUR* expression. Gene expression (Gene/*GAPDH*) was determined using the delta-delta CT method. Relative expression was calculated when the average gene expression (Gene/*GAPDH*) level in MC-PF was 1.

### 4.4. Statistical Analyses

Statistical analyses were conducted using SPSS 25.0. All data were tested for normality using Shapiro–Wilk’s test. The Kruskal–Wallis test was used to compare gene expression among the three BMI groups. To create a matched cohort of NW and OB OA patients, we calculated each individual’s propensity score based on the baseline clinical variables, age, and proportion with Kellgren and Lawrence grade 2–4. Analysis of categorical variables was performed using Fisher’s exact test; the relationship between *NMU*/*NMURs* and *CPA3* was determined using Spearman’s correlation coefficient; and comparison of gene expression between MC-RF and MC-PF was conducted using the Mann–Whitney U test. *p* < 0.05 was indicative of statistical significance.

## 5. Conclusions

In conclusion, *NMUR1* expression was increased in the SM of obese OA patients and its expression was found in MCs. A further investigation to analyze NMU/NMUR1 pathway in MC may provide a link between obesity and KOA pathology.

## Figures and Tables

**Table 1:** Patients’ clinical characteristics by body mass index group.

	Normal (*n* = 79)	Overweight (*n* = 87)	Obese (*n* = 40)	*p*
Age (years)	76.4 ± 6.5	72.8 ± 7.2 ^a^	72.1 ± 7.1 ^a^	0.004
KL (2/3/4), *n*	3/14/62	5/21/61	1/7/32	0.675
BMI (kg/m^2^)	22.3 ± 1.8	27.3 ± 1.5 ^a^	33.2 ± 2.6 ^a,b^	<0.001

KL, Kellgren/Lawrence grade; BMI, body mass index. Data are mean ± standard deviation (SD) unless otherwise indicated. ^a^ *p* < 0.05 compared with the normal group, ^b^ *p* < 0.05 compared with the overweight group.

**Table 2:** Clinical characteristics after propensity score analysis.

	Normal (*n* = 38)	Obese (*n* = 38)	*p*
Age (years)	74.4 ± 7.2	72.9 ± 6.2	0.429
KL (2/3/4), *n*	2/11/25	1/7/30	0.432
BMI (kg/m^2^)	32.9 ± 2.4	22.2 ± 2.1	<0.001

KL, Kellgren/Lawrence grade; BMI, body mass index. Data are mean ± standard deviation (SD) unless otherwise indicated.

**Table 3:** Sequences of primers used in this study.

Primer	Sequence (5’–3’)	Product Size (bp)
*CPA3*-F	GGCACTGACCTCAACAGGAA	71
*CPA3*-R	TCTGCACATGGGTCATTGGT	
*NMU*-F	GAGATGCTGCGAACAGAGAG	126
*NMU*-R	TATTGGAGCACCTCGGCAG	
*NUMR1*-F	ATGCTGTTTGTCCTGGTCGT	140
*NMUR1*-R	AAGATGCCGGAGATGACGTG	
*NMUR2*-F	TGAAGACGCCCACCAACTAC	165
*NMUR2*-R	AGCACACGGTCTCAAAGAGG	
*GAPDH*-F	TGTTGCCATCAATGACCCCTT	202
*GAPDH*-R	CTCCACGACGTACTCAGCG	

## Data Availability

The data presented in this study are available on request from the corresponding author.
